# Intestinal tuberculosis in a patient with end-stage renal disease on hemodialysis: A case report

**DOI:** 10.1097/MD.0000000000021641

**Published:** 2020-08-07

**Authors:** In Hee Lee, Seong Gyu Kim, Joong Goo Kwon, Chun-Seok Yang, Sungmin Kang, Min-Kyung Kim, Dong Jik Ahn

**Affiliations:** a Department of Internal Medicine; b Department of General Surgery; c Department of Nuclear Medicine, Daegu Catholic University School of Medicine, Daegu; d Department of Pathology, Dongguk University College of Medicine, Gyeongju; e Department of Internal Medicine, HANSUNG Union Internal Medicine Clinic and Dialysis Center, Daegu, Republic of Korea.

**Keywords:** tuberculosis, end-stage renal disease, hemodialysis, colon cancer

## Abstract

**Rationale:**

Intestinal tuberculosis (TB) is rarely seen in patients with end-stage renal disease (ESRD). We report an intestinal TB case with a clinical presentation similar to that of colon cancer in a patient with ESRD on hemodialysis.

**Patient concerns:**

A 49-year-old man presented with a 3-month history of general weakness and anorexia. He had been treated for stage 5 chronic kidney disease (CKD) due to diabetic nephropathy for the last 3 years. His blood urea nitrogen and serum creatinine levels were 96.9 and 8.1 mg/dL, respectively, at the time of admission; azotemia was accompanied by severe anemia, hypoalbuminemia, hyperkalemia, and metabolic acidosis. Hemodialysis was initiated for suspected exacerbation of uremia; however, intermittent fever, night sweats, and abdominal discomfort persisted.

**Diagnoses:**

Abdominal computed tomography (CT) and whole-body ^18^F-fluorodeoxyglucose positron emission tomography were indicative of ascending colon cancer with lymph node metastases. However, colonoscopy with biopsy revealed the formation of submucosal caseating granuloma and acid-fast bacillus.

**Interventions:**

We initiated quadruple therapy with isoniazid, rifampicin, pyrazinamide, and ethambutol. The patient continued the quadruple regimen for the first 2 months before switching to dual therapy and received anti-TB medications for a total of 12 months.

**Outcomes:**

After 9 months of standard anti-TB chemotherapy, polypoid residual lesions were noted during follow-up colonoscopy. Laparoscopy-assisted ileocecal resection was performed. No findings suggestive of recurrence of colonic TB were observed on follow-up abdominal CT at 6 months after discontinuation of anti-TB medications.

**Lessons:**

If non-specific uremic symptoms persist in patients with advanced CKD, the possibility of extrapulmonary TB such as intestinal TB must be considered. Also, in patients with radiologic suspicion of colon cancer, endoscopy with biopsy should be performed promptly to exclude colonic TB with similar clinical manifestations.

## 1 Introduction

The risk of *Mycobacterium tuberculosis* (*M tuberculosis*) infection is high in patients with chronic kidney disease (CKD) because of suppressed cell-mediated immunity resulting from various causes, such as advanced age, hypoalbuminemia, malnutrition, uremia, and immunosuppressive therapy.^[1]^ The prevalence of active tuberculosis (TB) in patients with end-stage renal disease (ESRD) undergoing dialysis is 6 to 25 times higher than that in the general population, which may increase the mortality rate of these patients.^[2]^ Extrapulmonary TB is a disease in which *M tuberculosis* invades many different organs other than the lung parenchyma, and patients with CKD infected with *M tuberculosis* more commonly present non-specific symptoms because the frequency of extrapulmonary TB is higher than that of pulmonary TB.^[3]^ Extrapulmonary TB mainly involves the lymph nodes, bones, peritoneum, and bone marrow in patients with ESRD, but intestinal TB has been rarely reported.^[4,5]^ Therefore, we report the case of a 49-year-old male patient who was on hemodialysis for ESRD secondary to diabetic nephropathy and was misdiagnosed with colon cancer before being confirmed as colonic TB via endoscopic biopsy.

## 2 Case report

**Figure 1:**
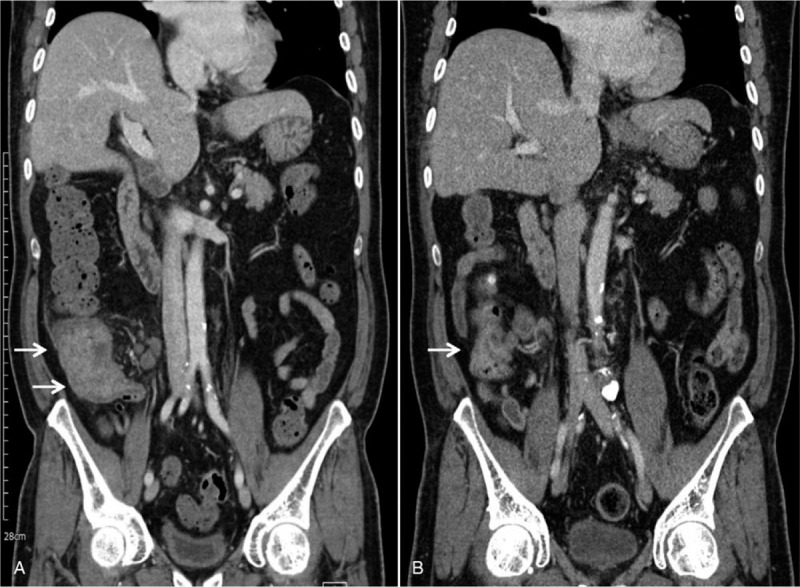
Abdominal computed tomography (CT) findings. (A) Initial abdominal CT scan shows a mass which is located just distal to ileocecal valve (arrows), with the formation of adjacent multiple lymphadenopathy. (B) Abdominal CT obtained at 9 months after antituberculous chemotherapy shows marked resolution of colonic tuberculosis (arrow). CT = computed tomography.

**Figure 2:**
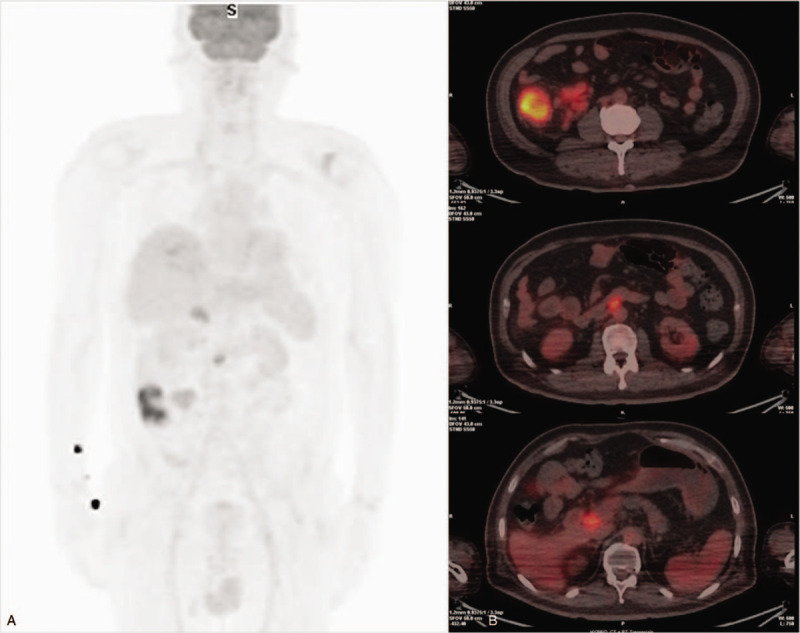
(A and B) ^18^F-fluorodeoxyglucose (FDG) positron emission tomography/computed tomography at presentation shows wall thickening with increased ^18^F-FDG uptake (maximum standardized uptake value 13.55) in ascending colon and enlarged lymph nodes with increased ^18^F-FDG uptake (7.09) in pericolic, aortocaval, and porta hepatis areas. FDG = fluorodeoxyglucose.

**Figure 3:**
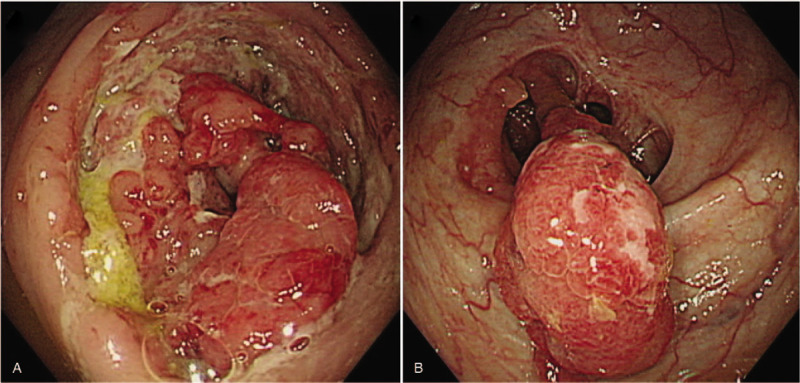
Colonoscopic findings. (A) Initial colonoscopy shows a large fungating circumferential mass with hypertrophic ulcerations in the ascending colon. (B) Semi-pedunculated polypoid masses of reduced size are noted on the 9-month follow-up colonoscopy.

**Figure 4:**
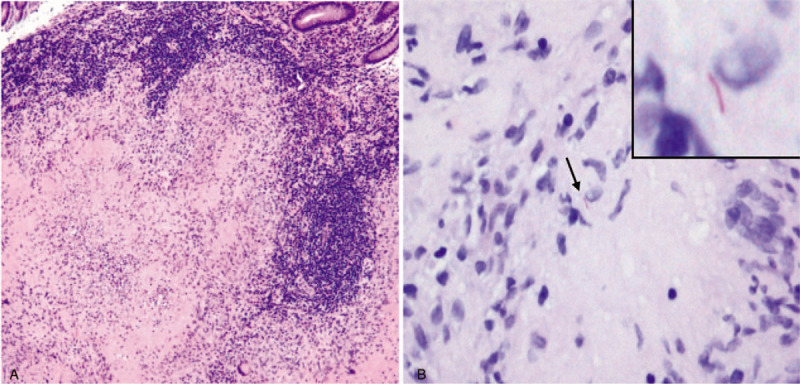
Microscopic features of colon specimen. (A) Hematoxylin and eosin stain shows confluent granulomatous inflammation with caseous necrosis below muscularis mucosa. Lymphocyte cuffing around the granulomas is also seen (×100). (B) Acid-fast bacillus stain demonstrates mycobacterium as a red rod (arrow) in the interface of caseous necrosis and viable cells (×400).

A 49-year-old male patient was admitted to our hospital with a 3-month history of general weakness and anorexia. The patient had been treated for stage 5 CKD secondary to diabetic nephropathy at the nephrology division for the last 3 years. The patient's blood urea nitrogen (BUN) level was 57.2 mg/dL and creatinine (Cr) level was 6 mg/dL at 3 months before admission, and he reported worsening of uremic symptoms, such as malaise, weight loss, anorexia, nausea, and vomiting. After his diagnosis of type 2 diabetes mellitus 20 years before admission, the patient was on combination therapy including oral hypoglycemic agents and insulin. He had no past history of pulmonary TB and viral hepatitis. At the time of admission, his blood pressure, pulse rate, respiration rate, and body temperature (BT) were 109/53 mm Hg, 80 beats/min, 20 breaths/min, and 37.3 °C, respectively. His consciousness was clear, and no murmur or crackles were detected on chest auscultation, although both conjunctivae were pale. No signs of a mass, organomegaly, and nearby tenderness were observed on the abdominal examination. A peripheral blood test on admission revealed the following: white blood cell (WBC) count, 7000/μL (neutrophils, 72%); hemoglobin, 5.2 g/dL; platelets, 374,000/μL; and erythrocyte sedimentation rate, 44 mm/h. Serum biochemical examination revealed the following: glucose, 267 mg/dL; total protein, 6.2 g/dL; albumin, 2.6 g/dL; BUN, 96.9 mg/dL; Cr, 8.1 mg/dL (estimated glomerular filtration rate, 8 mL/min); aspartate aminotransferase, 7 IU/L; alanine aminotransferase, 18 IU/L; Na^+^/K^+^/Cl^−^/total CO_2_, 130/6.0/100/14 mEq/L; calcium, 7.6 mg/dL; phosphorus, 6.2 mg/dL; uric acid, 13.4 mg/dL; and C-reactive protein, 14.6 mg/L. On urinalysis, 2+ was observed for albumin and 1+ for occult blood, and microscopic urinary sediment evaluation revealed 1 to 3 WBCs per high-power field (HPF) and 3 to 5 red blood cells per HPF. Serum immunological tests showed the following: iron, 14 μg/dL; total iron binding capacity, 199 μg/dL; ferritin, 172 ng/mL; and HbA_1_C, 8%. A 24-h urine examination revealed a urinary protein excretion level of 1156 mg/day and Cr clearance of 8.8 mL/min/1.73 m^2^. The patient's chest radiograph did not reveal pulmonary infiltrates in either lung field. On admission, hemodialysis was initiated after insertion of a dual-lumen temporary catheter for suspected exacerbation of uremia. Regular hemodialysis was conducted three times a week, and an autologous arteriovenous fistula was created on the 10th hospital day. During hemodialysis on the 14th day of hospitalization, high fever with a BT of 38.3°C was noted. With suspicion of catheter-related infection, we exchanged the catheter used for vascular access for hemodialysis and administered cefazolin (2.0 g, three times per week) after each hemodialysis. Methicillin-sensitive *Staphylococcus epidermidis* was isolated from blood cultures. On the 25th day of hospitalization, the patient presented with intermittent low-grade fever, abdominal pain, and watery diarrhea. Polymerase chain reaction (PCR) for *Clostridium difficile* toxins in stool specimens detected the toxin B-positive strain. On the diagnosis of pseudomembranous colitis (PMC), we discontinued the administration of cefazolin and administered oral metronidazole (1500 mg/day). However, the patient's abdominal discomfort, fever (BT, 37.5–38.3°C), and night sweats persisted. Because the origin of fever was not clear, abdominal computed tomography (CT) performed on the 28th hospital day revealed masses with irregular walls in the proximal portion of the ascending colon near the ileocecal valve and adjacent lymphadenopathy (Fig. 1A); an increase in ^18^F-Fluorodeoxyglucose (FDG) uptake was also noted in the corresponding region on whole-body positron emission tomography (PET)/CT scan (Fig. 2). The radiologic diagnosis was colon cancer with lymph node metastases. Colonoscopy with biopsy for histopathologic diagnosis was performed. A fungating circumferential mass with hypertrophic ulcerations, which mimicked a colonic tumor, was found in the ascending colon (Fig. 3A). However, microscopic examination demonstrated chronic caseating granulomatous inflammation and positive Ziehl-Neelsen stain for acid-fast bacillus (AFB), consistent with colonic TB (Fig. 4A and B). PCR analysis of the colonic lesions was negative for *M. tuberculosis*. The patient's tuberculin skin test (TST) result was also negative, and tumor markers, such as carcinoembryonic antigen and carbohydrate antigen 19-9, were all normal. We recommended surgical intervention including hemi-colectomy in view of the morphologic characteristics of the colonic lesions, but the patient refused surgery. Accordingly, we initiated quadruple therapy (HRZE): isoniazid (INH; 300 mg/day), rifampicin (RIF; 600 mg/day), pyrazinamide (PZA; 30 mg/kg, three times per week), and ethambutol (EMB; 15 mg/kg, three times per week). The patient was discharged from the hospital on the 45th day of admission after complete resolution of systemic symptoms. Anti-TB quadruple therapy was applied for the first 2 months; thereafter, it was switched to dual combination therapy (HR) of INH and RIF. Three months after the administration of anti-TB medications, follow-up colonoscopy showed improvement in colonic lesions. However, 9 months after the initiation of quadruple therapy, a second follow-up abdominal CT and colonoscopy showed polypoid residual lesions in the ascending colon (Figs. 1B and 3B) and we conducted laparoscopy-assisted ileocecal resection. Anti-TB chemotherapy was maintained for a total of 12 months. Progression of lymphadenopathy in the abdominal cavity or recurrence of colonic TB was not noted on follow-up abdominal CT performed at 6 months after discontinuation of anti-TB medications.

## 3 Discussion

As renal function declines, immune deficiencies deteriorate in patients with CKD because of various clinical factors, such as oxidative stress, chronic inflammation, 25(OH)-vitamin D deficiency, malnutrition, and secondary hyperparathyroidism.^[6]^ In particular, patients with CKD have a higher TB risk, increased reactivation of latent TB, and worse progression of active TB than the general population owing to the impairment of the cell-mediated immune system, which is involved in eliminating intracellular microorganisms such as *M tuberculosis.*^[7]^ Old age, male sex, diabetes mellitus, hypoalbuminemia, low body mass index, chronic anemia, ischemic heart disease, and smoking have been reported as significant independent risk factors for the development of TB in patients with ESRD on hemodialysis.^[8]^ TB is most commonly seen at the early stage of dialysis, particularly within 1 year after hemodialysis initiation, when malnutrition worsens and cell-mediated immunity greatly decreases.^[7]^ Thereafter, the frequency of TB tends to decrease as the patient's immunity improves with adequate dialysis therapy, although active TB itself is considered to be a major risk factor for increased morbidity and mortality in patients with ESRD.^[7]^ Because the incidence of extrapulmonary TB is as high as 60% to 80% in patients with CKD, various non-specific symptoms appear at the time of onset of TB, which may lead to delay in early diagnosis and appropriate treatment.^[2,3]^ In the analysis of 70 cases of extrapulmonary TB in patients with ESRD on maintenance dialysis, Yang et al reported that the mean age was 51.4 ± 17.8 years, and the male-to-female distribution was 0.9:1. They also described that the peritoneum (31.4%) was the major organ that developed extrapulmonary TB, followed by the bone, lymph nodes, bone marrow, spleen, and liver.^[9]^ In addition, fever (58.6%), pain, and lymphadenopathy were the most common clinical findings at the presentation of extrapulmonary TB, but non-specific symptoms, such as weight loss, anorexia, vomiting, and chronic anemia, were also often reported.^[9]^

Gastrointestinal TB comprises 3% to 5% of the total extrapulmonary TB cases in the general population,^[10]^ but intestinal TB is rare in patients with CKD.^[5]^ Intestinal TB can be caused by the reactivation of a primary infection. In addition, patients with active pulmonary TB may develop intestinal TB through various routes, such as swallowing infected sputum, hematogenous dissemination of AFB in patients with active pulmonary or miliary TB, consumption of food or milk contaminated with *M tuberculosis*, and spreading of infection directly from infected adjacent organs or lymph nodes.^[10]^ Intestinal TB is commonly observed in the ileocecal region, and hypertrophic lesions similar to polyps or tumors, segmental ulcerations, enterocolitis, and, rarely, diffuse colonic inflammation occur when *M tuberculosis* invades the gastrointestinal tract, which can cause the development of abdominal masses, local tenderness, and abdominal distention. Chronic abdominal pain is the most common symptom of intestinal TB, but it can also be accompanied by non-specific symptoms, such as unexplained fever, weight loss, nausea, vomiting, diarrhea, and bloody stools. Therefore, it is important to make a differential diagnosis from other gastrointestinal diseases, such as malignant tumors, Crohn's disease, intestinal lymphoma, *Yersinia* infection, and amoebic enteritis.^[11]^

Our patient had been undergoing conservative management for advanced CKD for several years and complained of general weakness, anorexia, nausea, and vomiting at the time of admission. Based on the laboratory findings and the patient's pre-existing diseases, hemodialysis along with a diagnosis of ESRD was recommended. After the initiation of hemodialysis, laboratory abnormalities and systemic symptoms improved, but fever developed 2 weeks after the start of hemodialysis. We conducted empirical antimicrobial therapy for catheter-related infections. Thereafter, PMC also developed; accordingly, metronidazole was administered orally and intravenous antibiotics were discontinued. However, low-grade pyrexia, night sweats, and abdominal discomfort continued, and ascending colon cancer with distant lymph node metastases was suggested based on the results of abdominal CT and whole-body PET/CT (Fig. 1A, 2). When colonic disease is suspected, radiologic examinations such as barium colonography, abdominal CT, and abdominal magnetic resonance imaging are recommended. However, it is not easy to confirm colonic TB with these studies.^[10]^ Gross identification of intestinal lesions by colonoscopy, microscopic analysis, and culture of the colonic tissue are the most useful diagnostic procedures for colonic TB.^[10]^ PCR analysis of intestinal mucosa is another adjunctive diagnostic method for confirming TB and has been reported to be more sensitive than tissue culture or AFB staining.^[10]^ TST is widely used as an additional diagnostic tool for TB, but its usefulness is limited because of immune abnormalities in patients with ESRD undergoing dialysis.^[12]^ Because active or past pulmonary TB is accompanied only in 25% of patients with extrapulmonary TB, intestinal TB cannot be excluded even if chest radiography results are negative.^[3]^ In our case, abnormal findings suggestive of pulmonary TB scarring or active TB lesion were not found on the chest radiography. PCR and TST of the patient's colonic mucosa also showed negative results. However, as formation of caseating granulomas and positive results on AFB staining were identified on microscopic examination of the colon specimen, he was diagnosed with colonic TB (Fig. 4A and B). In this patient, it was postulated that male sex, diabetes mellitus, chronic anemia, hypoalbuminemia, and malnutrition were significant risk factors of colonic TB. However, the definitive diagnosis of colonic TB and treatment initiation were delayed because of non-specific symptoms, catheter-related bacteremia that occurred after the initiation of hemodialysis, and PMC.

For the primary treatment of intestinal TB in patients with CKD, standard chemotherapy, that is, quadruple therapy (HRZE) for the first 2 months followed by dual therapy (HR) for a further period of 4 to 7 months thereafter, is recommended.^[13]^ However, administration of anti-TB medications is often continued for a period longer than 9 months and, in particular, up to 12 to 18 months.^[14,15]^ Patients with CKD are more susceptible to treatment failure of TB because of a higher incidence of adverse effects related to anti-TB medications and poor adherence to medications as well as immune system abnormalities. Therefore, adequate dosing intervals and careful follow up are required.^[16,17]^ Surgical intervention should be considered in addition to anti-TB drug therapy if there is bowel perforation with or without abscess or fistula, massive gastrointestinal bleeding, complete bowel obstruction, or intestinal obstruction with no response to anti-TB medications.^[11]^ There were no medication-related toxicities or specific side effects observed in our patient during the 12-month standard anti-TB therapy. In contrast, his systemic symptoms such as fever and abdominal pain were markedly resolved after the initiation of anti-TB medications, and gradual improvement in colonic lesions was demonstrated in the follow-up colonoscopy. However, ileocecal resection was additionally performed because residual lesions were noted in the ascending colon even after 9 months of anti-TB chemotherapy (Fig. 3B). It has been reported that colon cancer might develop later because of chronic inflammation and abnormal immune responses at the site of a previous intestinal TB lesion.^[18,19]^ Therefore, appropriate surgical intervention is needed for residual lesions after completion of anti-TB chemotherapy.

In summary, in our patient with stage 5 CKD secondary to diabetic nephropathy, hemodialysis was initiated for suspected exacerbation of uremia. Colon cancer with lymph node metastases was suggested in the radiologic examinations, but colonic TB was documented by colonoscopic biopsy. In addition, our patient was treated with standard anti-TB chemotherapy for 12 months and underwent ileocecal resection. Based on the findings of this case, if non-specific uremic symptoms persist in patients with advanced CKD, the possibility of extrapulmonary TB must be considered in addition to conducting dialysis therapy. Also, in patients with radiologic suspicion of colon cancer, endoscopy with biopsy should be performed promptly to exclude colonic TB with similar clinical manifestations.

## Author contributions

**Conceptualization:** In Hee Lee.

**Data curation:** Joong Goo Kwon, Chun-Seok Yang, Sungmin Kang.

**Formal analysis:** In Hee Lee.

**Methodology:** In Hee Lee, Seong Gyu Kim.

**Validation:** Min-Kyung Kim, Dong Jik Ahn.

**Writing – original draft:** In Hee Lee, Seong Gyu Kim.

**Writing – review & editing:** In Hee Lee.

In Hee Lee orcid: 0000-0003-3562-7586.
